# Intraoperative Angiographie bei A.-carotis-Rekonstruktion – pathologische Befunde, Zuverlässigkeit und Bedeutung des Verfahrens

**DOI:** 10.1007/s00104-021-01403-y

**Published:** 2021-04-14

**Authors:** Jasmin Dillner, Frank Meyer, Zuhir Halloul, Michael Görtler

**Affiliations:** 1 Klinik für Allgemein‑, Viszeral‑, Gefäß- und Transplantationschirurgie, Universitätsklinikum Magdeburg A.ö.R., Leipziger Straße 44, 39120 Magdeburg, Deutschland; 2 Klinik für Neurologie, Universitätsklinikum Magdeburg A.ö.R., Leipziger Straße 44, 39120 Magdeburg, Deutschland

**Keywords:** A.-carotis-Desobliteration, Duplexsonographie, Postoperatives Komplikationsprofil, Outcome, Morbidität, Carotid artery occlusion, Duplex ultrasonography, Postoperative complication profile, Outcome, Morbidity

## Abstract

**Ziel:**

Der Wert der intraoperativen Angiographie und deren Ad-hoc-Beurteilung im Hinblick auf operationstechnische Unzulänglichkeiten soll geprüft werden.

**Methode:**

Insgesamt 523 konsekutive A.-cartotis-Thrombendarteriektomie(TEA)-Patienten mit intraoperativer Kontrollangiographie, postoperativer Duplexsonographie und retrospektiver Zweitbeurteilung der Angiographie wurden in die Auswertung einbezogen.

**Ergebnisse:**

In der retrospektiven Zweitbeurteilung der Angiographie wurden 23 (4,4%) Verschlüsse oder hochgradige Stenosen der Arteria carotis communis (ACC) oder Arteria carotis interna (ACI) im Operationsbereich (12/2,3%) bzw. in der Abstrombahn (distale extrakranielle und intrakranielle ACI, A. cerebri media [ACM]) detektiert (11/2,1%), wohingegen bei der intraoperativen Ad-hoc-Beurteilung nur 13 (2,5%) derartige Pathologien beschrieben worden waren (7/1,3% im Operationsbereich, 6/1,1% in der Abstrombahn; *p*=0,002). Duplexsonographisch wurden postoperativ bei 50 von 505 untersuchten Patienten (10,1%) operationstechnische Unzulänglichkeiten lokal detektiert, was signifikant mehr war als in der Angiographie (*p*<0,001). In den meisten Fällen handelte es sich um nicht okkludierende/nicht hochgradig stenosierende Intima-Media-Ablösungen (19), Nahteinziehungen (13) und Kinkings/Kalibersprünge am distalen Patchende (14). Nahteinziehungen und Kinkings/Kalibersprünge waren mit einer linksseitigen TEA (adjustierte OR: 2,4; 95%-KI: 1,1‑5,1), einer Operation ohne Patch (adjustierte OR: 16,6; 95%-KI: 1,3–215,0) und der Verwendung eines Dacron- vs. Polytetrafluorethylen-Patch (adjustierte OR: 3,0; 95%-KI: 1,4–6,6) assoziiert.

**Schlussfolgerung:**

Bei der Ad-hoc-Beurteilung der intraoperativen Kontrollangiographie kann eine nicht unerhebliche Zahl auch okkludierender und hochgradig stenosierender Pathologien übersehen werden (zur Detektion nicht okkludierender und nicht hochgradig stenosierender operativer Unzulänglichkeiten methodisch nicht geeignet – Ausweich: postoperative Duplexsonographie).

## Abstract (en)

**Aim:**

To investigate the value of intraoperative angiography and its ad hoc evaluation with respect to cases of surgical technical inaccessibility.

**Methods:**

Overall, 523 consecutive carotid artery thrombendarterectomy (TEA) patients with intraoperative control angiography, postoperative color-coded duplex sonography and retrospective re-evaluation of documented angiographic images were included in the evaluation.

**Results:**

In the retrospective angiographic re-evaluation 23 (4.4%) occlusions or high-grade stenoses of the common carotid artery (CCA) or internal carotid artery (ICA) in the surgical field (12, 2.3%) or of downstream ICA or middle cerebral artery (MCA, 11, 2.1%) were detected. The detection rate was significantly lower in the intraoperative ad hoc evaluation with overall only 13 (2.5%) detected pathologies (7, 1.3% in the surgical field, 6, 1.1% in large downstream arteries, *p*=0.002). Postoperative duplex sonography performed in 505 patients detected 50 cases (10.1%) of local surgical technical inaccessibility, which was significantly more than in the angiography (*p*<0.001). In most cases these were nonocclusive, low-grade stenosing detachments of the intima media (*n*=19), 13 suture contractions, and 14 kinking/abrupt diameter changes at the distal end of the patch. Suture contractions and kinking/diameter changes were associated with a left-sided TEA (adjusted odds ratio, OR 2.4, 95% confidence interval, CI 1.1–5.1), an operation without a patch (adjusted OR, 16.6, 95% CI 1.3–215.0), and using Dacron patches in contrast to PTFE patches (adjusted OR 3.0, 95% CI 1.4–6.6).

**Conclusion:**

The ad hoc evaluation of intraoperative completion angiography by surgeons missed a substantial number also of occluding and severely stenosing pathologies. Angiography is not suitable for the detection of nonocclusive and low-grade stenosing cases of operative inaccessibility. Postoperative color-coded duplex sonography is an adequate tool for surgical quality control.

## Hintergrund

Die Thrombendarteriektomie (TEA) einer A.-carotis-Stenose ist eine etablierte Behandlung, deren Indikation anhand zahlreicher randomisierter Studien gut untersucht ist [6, 7, 11, 12]. Aufgrund des primär prophylaktischen Effekts der Operation kommt der Relation zwischen zukünftigem Nutzen und aktuellem Eingriffsrisiko bei der Patientenauswahl eine entscheidende Bedeutung zu [3]. Das Eingriffsrisiko wird, neben dem allgemeinen Operations- und Anästhesierisiko, wesentlich durch das Risiko eines perioperativ induzierten Schlaganfalls bestimmt. Etwa die Hälfte derartiger Schlaganfälle wird dabei auf Thrombembolien zurückgeführt, deren Ursprung der Operationsbereich ist, und von denen wiederum etwa 80 % operationstechnischen Unzulänglichkeiten zugeschrieben werden [16]. Zur Reduktion derartiger Komplikationen wird vielfach eine sich unmittelbar an die Rekonstruktion anschließende intraoperative Angiographie mit ggf. unmittelbarer operativer Korrektur propagiert [15, 22] Allerdings konnte deren Nutzen im Hinblick auf eine Reduktion perioperativer Schlaganfälle in dazu durchgeführten Untersuchungen – bisher ausschließlich mittels serieller Vergleichsgruppen – nicht nachgewiesen werden [4, 9, 17, 23]. Ein Grund dafür dürfte sein, dass kein Konsens besteht, welche technischen Unzulänglichkeiten relevant und korrekturbedürftig sind [5, 13, 24]. Weitere mögliche Gründe könnten eine zu geringe Sensitivität der intraoperativen Angiographie für einen pathologischen Befund sein und/oder eine nicht ausreichende Sensitivität der Ad-hoc-Beurteilung durch den Operateur [5, 18].

Ziel der hier vorgestellten Studie war, die beiden letztgenannten Aspekte zu untersuchen und zu quantifizieren. Dazu wurden (1) intraoperativ durchgeführte Angiographien von einem unabhängigen Gefäßchirurgen nachbeurteilt sowie (2) ein prospektiv 3 bis 5 Tage postoperativ erhobener duplexsonographischer Befund mit der angiographischen Nachbeurteilung verglichen. Darüber hinaus wurden klinische und operative Variablen auf ihre Assoziation mit operativ-technischen Unzulänglichkeiten überprüft.

## Patienten, Material und Methoden

### Patientenauswahl

Alle Patienten, die sich von 1995 bis 2012 in der Abteilung Gefäßchirurgie der Universitätsklinik für Allgemein‑, Viszeral‑, Gefäß- und Transplantationschirurgie am Universitätsklinikum Magdeburg A.ö.R. der TEA einer A.-carotis-Stenose unterzogen und bei denen im Anschluss an die Rekonstruktion intraoperativ eine angiographische Kontrolle erfolgte, wurden für diese Studie ausgewählt. Seit 2004 wurde diese Kontrolle routinemäßig bei jeder TEA durchgeführt, davor war dies nur sporadisch der Fall. Alle diagnostischen und therapeutischen Maßnahmen erfolgten im Rahmen des klinischen Behandlungsstandards, die Datendokumentation und -speicherung fand nach den Regeln nationaler und internationaler Datenschutzverordnungen statt. Die zuständige Ethikkommission der Otto-von-Guericke-Universität Magdeburg verneinte daher die Notwendigkeit einer Patientenzustimmung für die retrospektive Datenauswertung. Die Studie wurde im Deutschen Register Klinischer Studien (DRKS) unter der Studiennummer DRKS00023151 registriert.

### Präoperative Untersuchungen

Die standardisierte präoperative Diagnostik umfasste die Anamnese, den neurologischen Status, ein zerebrales Computertomogramm (CT)/Magnetresonanztomogramm (MRT), Laboruntersuchungen und ein Elektrokardiogramm (EKG). Der extra- und intrakranielle Gefäßstatus wurde mittels Doppler- und farbkodierter Duplexsonographie erhoben. Im Fall eines unklaren sonographischen Befundes oder eines fehlenden temporalen Schallfensters zur Beurteilung der intrakraniellen Gefäße erfolgte zusätzlich eine MR- oder CT-Angiographie. Der Stenosegrad der A. carotis interna (ACI) wurde duplexsonographisch nach publizierten Kriterien bestimmt [1] und als distale Durchmesserreduktion (NASCET [North American Symptomatic Carotid Endarterectomy Trial]-Stenosegrad) in 10 %-Schritten angegeben. Bei Patienten mit einem temporalen Schallfenster wurde zusätzlich die zerebrovaskuläre Reservekapazität mittels Doppler-CO_2_-Test bestimmt [8, 21].

### Anästhesie- und Operationsverfahren

Die TEA wurde routinemäßig als Patch-Endarteriektomie intendiert und erfolgte bis 2001 in Allgemeinanästhesie unter Einsatz eines Shunts. Seit 2002 kam eine Regionalanästhesie mit kombinierter superfizieller und tiefer zervikaler Plexusanästhesie zur Anwendung. Vor dem Abklemmen der A. carotis wurde Heparin intravenös appliziert und am Operationsende mittels Protaminsulphat in Höhe der halben applizierten Heparindosis antagonisiert. Bei der Operation in Regionalanästhesie wurde die Notwendigkeit eines Shunts klinisch überprüft (Drücken einer „Quietsch-Ente“ mit der Hand kontralateral zur Operationsseite). Im Weiteren erfolgten die Längsarteriotomie und die direkte Desobliteration. Bei sichtbarer Intimastufe nach distal wurde diese fixiert und im Anschluss die Längsarteriotomie mittels Patchplastik verschlossen. Nach Beendigung der Patchnaht erfolgte die intraoperative Angiographiekontrolle durch einen C‑Bogen (OEC 9600/9800, General Electric; seit 2009 Arcadis Avantic, Siemens, München, Deutschland) mittels Punktion der A. carotis communis (ACC) proximal des eingenähten Patches und Injektion von 5–10 ml jodhaltigem Kontrastmittel (Imeron 300, Bracco) über eine 22-Gauche-Butterfly-Kanüle. Standardmäßig erfolgte eine Aufnahme in 30°-lateraler Projektion, bei unklarer Situation ggf. erweitert um eine a.p. oder laterale Projektion bzw. nochmalige Injektion. Bei unauffälligen Perfusionsverhältnissen im Patchbereich und in der Abstrombahn wurde die Operation beendet. Postoperativ wurde der Patient für 24 h auf der Stroke-Unit überwacht.

### Intraoperative DSA und postoperative Duplexsonographie

Die intraoperative Angiographie wurde vom Operateur vorgenommen und in Form eines oder mehrerer Bilder dokumentiert (Print, seit 2009 digital). Die Beurteilung erfolgte durch ihn bzw., im Fall eines weniger erfahrenen Chirurgen, durch den in derartigen Fällen mitoperierenden und supervidierenden Gefäßchirurgen. Als Indikation für einen chirurgischen Korrektureingriff galten pathologische Befunde i.S. von Stenosen > 60 % und ein Verschluss im Bereich der endarteriektomierten ACC und ACI. Neu eingetretene Verschlüsse nur in der Abstrombahn stellten eine Indikation für eine unmittelbar postoperative endovaskuläre Thrombektomie dar. Korrektureingriffe bei ACE-Pathologien wurden vom Operateur entschieden, für hochgradige Stenosen in der Abstrombahn erfolgte dies ad hoc gemeinsam gefäßchirurgisch und neurologisch. Die 3 bis 5 Tage postoperativ – bei (neuer) postoperativer neurologischer Symptomatik umgehend – durchgeführte Duplexsonographie erfolgte durch einen DEGUM-zertifizierten Neurologen, dem die angiographischen Befunde und ein möglicher Korrektureingriff nicht bekannt waren. Lokalbefund (ACC; ACI; A. carotis externa, ACE) und Abstrombahn (extrakranielle distale und intrakranielle ACI; A. cerebri media, ACM) wurden duplexsonographisch jeweils getrennt als pathologisch/nichtpathologisch beurteilt. Im pathologischen Fall erfolgte eine Quantifizierung des Ausmaßes des pathologischen Befundes (Verschluss, hochgradige Stenose, nicht hochgradig stenosierend) und eine Einschätzung der (vermeintlichen) Ätiologie (Thrombus/Thrombembolie, Nahteinziehung, Intima-Media-Ablösung, langstreckig enges Gefäß, residualer Plaque, Kalibersprung/Kinking am Übergang des Patches zur distalen ACI, extravasales Hämatom). Die Zweitbeurteilung der dokumentierten digitalen Subtraktionsangiographien wurde durch einen gefäßchirurgischen Facharzt mit TEA-Erfahrung retrospektiv im Anschluss an den Patienteneinschlusszeitraum vorgenommen, jeweils ohne Kenntnis der intraoperativen Befundung, einer möglicherweise stattgefundenen Korrekturoperation und des postoperativen duplexsonographischen Befundes. Die angiographische Zweitbeurteilung erfolgte analog zur Duplexsonographie.

### Klinisch-neurologisches Outcome

Bei einer ambulanten Wiedervorstellung des Patienten 6 Wochen postoperativ wurden mögliche Komplikationen, die erst poststationär aufgetreten waren, erfragt und der klinisch-neurologische Status erhoben. Patienten, die diesen Termin nicht wahrnahmen, wurden telefonisch kontaktiert. Die bis 4 Wochen postoperativ eingetretenen Befunde gingen in das perioperative klinisch-neurologische Outcome (persistierender Schlaganfall und Tod) ein.

### Vergleich der Untersuchungsverfahren und Prädiktoren für intraoperative Pathologika

Die Sensitivität der prospektiven und retrospektiven angiographischen Beurteilung und der prospektiven duplexsonographischen Untersuchung wurde für (1) Verschluss und hochgradige Stenose lokal im Operationsbereich (ohne ACE-Pathologie) und in der Abstrombahn sowie (2) jedwede Pathologie lokal im Operationsbereich (ohne ACE-Pathologien) untersucht. In einem zweiten Schritt wurde untersucht, ob klinische und operationstechnische Variablen mit dem Auftreten dieser operativen Pathologika assoziiert waren und inwieweit das Auftreten derartiger operativer Pathologika mit dem klinisch-neurologischen Outcome assoziiert war.

### Statistik

Die statistische Auswertung erfolgte mittels SPSS, Version 26 (SPSS Inc., Chicago/IL, USA). Der Vergleich zwischen den Untersuchungsmethoden erfolgte für die beiden angiographischen Auswertungen paarweise mittels McNemar-Test und bei Einbeziehung zusätzlich der Duplexsonographie mittels Cochran-Q-Test jeweils für verbundene Stichproben. Eine mögliche Assoziation zwischen klinischen und operationstechnischen Variablen und dem Auftreten operativer Pathologien sowie zwischen Letzteren und dem Auftreten eines perioperativen Schlaganfalls/Todes wurde mittels multivariater binärer logistischer Regression untersucht. Dabei wurden die unabhängigen Input-Variablen initial eingeschlossen und danach schrittweise rückwärts aus der Regression ausgeschlossen (*p*_Ausschluss_ ≥ 0,1; *p*_Wiedereinschluss_ < 0,05). Das Ergebnis für die in der Regressionsanalyse verbliebenen Variable wird als adjustierte Odds Ratio (OR) mit 95 %-Konfidenzintervall (KI) präsentiert. Als statistisch signifikant wurde eine Irrtumswahrscheinlichkeit von *p* < 0,05 (zweiseitig) festgelegt.

## Ergebnisse

Präoperativ waren 294 der 523 operierten Stenosen (56,2 %) symptomatisch. Bei 9 Patienten (1,7 %) bestand ein kurzstreckiger segmentaler Verschluss von der Bifurkation bis zum Abgang einer primitiven karotidovertebralen Anastomose. Bei 512 TEA handelte es sich um eine prophylaktische Erst- (509) oder Reoperation (3), bei 12 Patienten erfolgte der Eingriff im Rahmen eines akuten Schlaganfalls aufgrund einer zerebral-hämodynamischen Minderperfusion. 520 Stenosen (99,4 %) wurden in Form einer Patch-Endarteriektomie operiert, 2‑mal erfolgte eine Eversionsendarteriektomie, 1‑mal eine Direktnaht. Als Patchmaterial kamen bei 215 Operationen Dacron (41,1 %), bei 296 Polytetrafluoethylen (PTFE; 56,6 %), bei 8 bovines Perikard (1,5 %) und bei einer TEA eine autologe Vene zum Einsatz. Weitere Patientencharakteristika, klinische und Operationsdaten sind in Tab. 1 aufgeführt.

**Table 1:** 

Demographische, klinische und operative Daten	*n* (%)
Alter (Mittelwert [Standardabweichung], Jahre)	67,8 (9,5)
Männer	386 (73,8)
Arterielle Hypertonie	468 (89,5)
Diabetes mellitus	199 (36,0)
Koronare Herzkrankheit	183 (35,0)
*Symptomatik:*	
Asymptomatisch	229 (43,8)
Amaurosis fugax	50 (9,6)
TIA	57 (10,9)
Schlaganfall	187 (35,8)
*Stenosegrad (NASCET):*	
50 %	6 (1,1)
60 %	14 (2,7)
70 %	174 (33,3)
80 %	202 (38,6)
90 %	60 (11,5)
> 90 %	58 (11,1)
100 %	9 (1,7)
Kontralateraler Verschluss/hochgradige Stenose	111 (21,2)
Aufgehobene zerebrovaskuläre Reservekapazität	43 (8,2)
Regionalanästhesie (vs. Allgemeinanästhesie)	443 (84,7)
TEA-Seite rechts	253 (48,4)
Rechtshändigkeit des Erstoperateurs	523 (100)
*TEA-Frequenz Erstoperateur*	
< 20 Operationen	67 (12,8)
< 50 Operationen	42 (8,0)
≥ 50 Operationen	111 (21,2)
≥ 50 Operationen und ≥ 20 Operationen/Jahr	303 (57,9)
Shunt	142 (27,2)
Intimafixierung distal	480 (91,8)
*Präoperative TAH*	
Keine TAH	64 (12,2)
1 TAH	353 (67,5)
2 TAH	106 (20,3)

*TEA* Thrombendarteriektomie, *DSA* digitale Subtraktionsangiopathie, *TIA* transitorische ischämische Attacke, *TAH* Thrombozytenaggregationshemmer, *NASCET* North American Symptomatic Carotid Endarterectomy Trial

Perioperativ erlitten 21 Patienten (4,0 %) einen Schlaganfall, wovon 4 letal verliefen. Drei weitere Patienten starben 2, 9 bzw. 14 Tage postoperativ an einem Myokardinfarkt, ein Patient 3 Wochen nach der TEA an einer Pneumonie.

### Intraoperative DSA und Sofortkorrekturen

Intraoperativ wurden 21 Angiographien (4,0 %) als pathologisch beurteilt. Bei den 15 lokalen Pathologien (2,9 %) handelte es sich um 6 hochgradige Stenosen (1 in ACC, 5 in ACI) und 9 Verschlüsse (1 in ACI, 8 in ACE), von denen alle 6 hochgradige Stenosen, der ACI-Verschluss und 5 ACE-Verschlüsse korrigiert wurden (Sofortkorrekturrate lokal: 2,3 %). In der Abstrombahn wurden 3 Verschlüsse und 3 hochgradige Stenosen (1,1 %) neu gegenüber präoperativ beschrieben, von denen alle 3 Verschlüsse unmittelbar postoperativ endovaskulär thrombektomiert wurden. Die Ätiologie der 6 hochgradigen Stenosen im Operationsbereich war jeweils eine Nahteinziehung, die des lokalen ACI-Verschlusses und der 6 Pathologien in der Abstrombahn eine Thrombose bzw. Thrombembolie. ACE-Pathologien wurde keine Ätiologie zugeordnet, dies war anhand der Angiographie nicht ausreichend valide möglich (Tab. 2).

**Table 2:** 

	Operationsbereich lokal											
	ACC			ACI			ACE			Abstromgebiet		
	Vers	Hochgr	< Hochgr	Vers	Hochgr	< Hochgr	Vers	Hochgr	< Hochgr	Vers	Hochgr	< Hochgr
DSA intraoperativ	0	1	0	1	5	0	8	0	0	3	3	0
Ätiologie	–	1 NA	–	1 TH	5 NA	–	–	–	–	3 TH	3 TH	–
DSA retrospektiv	0	1 + 2	0	1	5 + 3	0	5	30	0	3 + 2	3 + 3	0
Ätiologie	–	1 NA + 2 TH^a^	–	T	5 NA + 3 UN	–	–	–	–	3 TH + 2 TH^b^	3 TH + 3 EN^c^	–
Duplexsonographie postoperativ	0	2	19	1	1	29	2	19	0	8	3 + 4	0
Ätiologie	–	2 IN	16 IN/3 RE	1 TH^a^	1 NA	12 NA/14 KI 2 HÄ/1 IN	–	–	A	8 TH^bc^	3 TH + 4 EN^b^	–

*ACC* A. carotis communis, *ACI* A. carotis interna, *ACE* A. carotis externa, *NA* Nahteinziehung, *TH* Thrombus/Thrombembolie, *UN* unbekannt, *EN* Engstellung, *IN* Intima-Media-Läsion, *RE* residualer Plaque, *KI* Kinking/Kalibersprung, *HÄ* perivaskuläres Hämatom

^a^Eine intraoperative thrombotische ACC-Stenose zu ACI-Frühverschluss progredient

^b^Ein intraoperativ thrombotischer Verschluss persistiert, eine zu hochgradigen Stenose mit Engstellung regredient

^c^Eine Engstellung zum Verschluss progredient, eine remittiert

Keiner der 15 Patienten mit einer lokalen Pathologie bot postoperativ eine (neue) neurologische Symptomatik oder hatte, sofern in Regionalanästhesie operiert (1 ACC-Stenose, 1 ACI-Verschluss, 3 hochgradige ACI-Stenosen, 8 ACE-Verschlüsse), intraoperativ eine Symptomatik geboten. Keiner der 3 Patienten mit einer hochgradigen Stenose in der Abstrombahn – jeweils in Regionalanästhesie operiert – hatte intra- oder postoperativ eine (neue) neurologische Symptomatik. Dagegen waren 2 der 3 Patienten mit einem Verschluss in der Abstrombahn postoperativ (neu) symptomatisch.

### Retrospektive DSA-Zweitbeurteilung

Zwei gegenüber intraoperativ zusätzlich detektierte hochgradige ACC-Stenosen waren im postoperativen Ultraschall zu einem asymptomatischen Frühverschluss (der ACI) progredient bzw. nicht mehr nachweisbar. Drei zusätzlich detektierte hochgradige ACI-Stenosen waren postoperativ ebenfalls nicht mehr nachweisbar und neurologisch nicht auffällig. In der Abstrombahn wurden zusätzlich zwei Verschlüsse detektiert, von denen einer symptomatisch war und persistierte, wohingegen der asymptomatische zu einer hochgradigen Stenose mit langstreckig enger distaler ACI partiell rekanalisierte. Zudem wurden in der Abstrombahn zusätzlich 3 hochgradige Stenosen in Form einer langstreckig engen distalen extrakraniellen ACI detektiert, von denen eine persistierte, eine remittierte und eine zum Verschluss progredient war, jeweils ohne neurologische Symptomatik.

### Postoperative Duplexsonographie

Von den 523 Patienten erhielten 3 keine postoperative Duplexsonographie. Die 15 intraoperativ sofort Rekonstruierten wurden ebenfalls duplexsonographisch untersucht, gingen aber nicht in die vergleichende Auswertung zwischen Angiographie und Duplexsonographie ein, da eine Befunddiskrepanz nicht nur methodisch, sondern durch den zwischenzeitlich durchgeführten Korrektureingriff erklärt werden kann. Duplexsonographisch wurden in der ACC 18 Intima-Media-Ablösungen (2 mit hochgradig stenosierendem Flap, eine davon symptomatisch) und 3 residuale Plaques detektiert, die angiographisch (intraoperativ/retrospektiv) nicht beschrieben wurden, darüber hinaus in der ACI am distalen Patchende 13 Nahteinziehungen (eine hochgradig stenosierend), 14 Kinking/Kalibersprünge am Übergang des Patches zum nichtoperierten Gefäßsegment, 2 extravasale Hämatome und eine Intima-Media-Ablösung, die alle nicht mit einer perioperativen Symptomatik assoziiert waren (Tab. 2). In der Abstrombahn wurden gegenüber der Angiographie (intraoperativ/retrospektiv) zusätzlich 6 Verschlüsse (2 neu symptomatisch) und 2 hochgradige Stenosen in Form einer langstreckig engen extrakraniellen distalen ACI detektiert (jeweils asymptomatisch; Tab. 2).

### DSA intraoperativ vs. retrospektiv vs. Duplex

Die Sensitivität für intraoperativ aufgetretene Pathologien in Form hochgradiger Stenosen und Verschlüsse (ohne ACE-Pathologien) war für die beiden DSA-Auswertungen signifikant unterschiedlich (*p* = 0,002; McNemar-Test). Fünf zusätzliche hochgradige Stenosen lokal im Operationsbereich – durch einen Thrombus (2-mal in ACC) bzw. wahrscheinlich durch einen Thrombus (3-mal in ACI) – und 5 in der Abstrombahn (2 Thrombembolien und 3 langstreckig enge Arterien) fielen erst bei der retrospektiven Nachbeurteilung der intraoperativen DSA auf, womit sich die Rate gegenüber der intraoperativen Beurteilung von 2,5 auf 4,0 % signifikant erhöhte. Die Häufigkeit beschriebener lokaler Pathologien nahm dabei von 1,3 auf 2,3 % zu, die von Pathologien in der Abstrombahn von 1,1 auf 2,1 %.

Der Vergleich aller drei Methoden erfolgte für jedwede lokale Pathologie der ACC und ACI (ohne ACE). Hierbei lag die Frequenz duplexsonographisch detektierter Pathologien signifikant über derjenigen, die in der DSA beschrieben worden waren (10,1 % vs. 2,3 %; *p* < 0,001, Chochran-Q-Test). Dies lag primär an der duplexsonographischen Detektion auch nicht okkludierender und nicht hochgradig stenosierender operativer Pathologien wie Intima-Media-Ablösungen in der ACC und Nahteinziehungen/Kinkings/Kalibersprüngen am distalen Patchübergang zur ACI (Tab. 2).

### Prädiktoren intraoperativer Pathologien

Lokale Thrombosen und oder Thrombembolien in der Abstrombahn waren assoziiert mit notfallmäßigen Eingriffen im Rahmen eines akuten Schlaganfalls (adjustierte OR: 96,8; 95 %-KI: 11,4–819,7), höchstgradigen (> 90 %) Stenosen (adjustierte OR: 5,8; 95 %-KI: 1,4–23,9) und einer fehlenden Thrombozytenaggregationshemmung präoperativ (adjustierte OR: 29,3; 95 %-KI: 1,9–442,6). Eine Assoziation mit lokalen Pathologien wie einer Intima-Media-Ablösung, einer Nahteinziehung und eines Kinkings/Kalibersprungs bestand nicht.

Eine Nahteinziehung, ein Kinking und/oder ein Kalibersprung in der Regel am distalen Patchübergang zur normalen ACI waren assoziiert mit einer TEA der linken ACI (adjustierte OR: 2,4; 95 %-KI: 1,1–5,1) sowie der Operation ohne Patch (adjustierte OR: 16,6; 95 %-KI: 1,3–215,0) bzw. unter Verwendung eines Dacron-Patches (adjustierte OR: 3,0; 95 %-KI: 1,4–6,6), letztere jeweils gegenüber der Verwendung eines PTFE-Patches.

### Intraoperative Pathologien und Outcome

Keine der lokalen Pathologien – weder in ihrer Gesamtheit, noch im Hinblick auf spezifische Ätiologien oder bei Betrachtung nur relevanter Ausprägungen (Verschluss/hochgradige Stenose) – war unabhängig mit dem Outcome assoziiert. Dies traf nur für Abstrompathologien zu (adjustierte OR 5,3; KI 1,2–24,7) und hier speziell für Thrombembolien (adjustierte OR 5,7; KI 1,2–27,7), die neben dem Alter (adjustierte OR pro Jahr 1,18, KI 1,02–1,12), der Verwendung eines Shunts (adjustierte OR 3,3; KI 1,2–8,7) und einem notfallmäßigen TEA-Eingriff (adjustierte OR 9,8; KI 1,8–53,1) ein Prädiktor für einen perioperativen Schlaganfall und Tod waren. Dies änderte sich auch nach Ausschluss der Patienten mit einem notfallmäßigen TEA-Eingriff und Betrachtung nur der sekundärpräventiv operierten Patienten nicht.

## Diskussion

In unserem Kollektiv wurden bei der Nachbeurteilung der Bilddokumentation der intraoperativen Kontroll-DSA 10 hochgradige Stenosen detektiert – 5 lokal im Operationsbereich an ACC bzw. ACI und 5 in der Abstrombahn, die durch den Operateur bei der intraoperativen Beurteilung nicht beschrieben worden waren. Da alle 13 intraoperativ beschriebenen derartigen Pathologien auch in der Nachbeurteilung erkannt wurden, lag die Sensitivität der unmittelbaren Beurteilung durch den Operateur nur bei 57 % und betrug die Rate detektierter derartiger Pathologien für die intraoperative DSA-Auswertung 2,5 % und für die retrospektive Auswertung 4,0 %. Dieser signifikante Unterschied scheint nicht alleine mit der spezifischen DSA-Durchführung und -Beurteilung in unserem Kollektiv zu erklären sein. So geben Studien, denen eine prospektive Auswertung der intraoperativen Kontrollangiographie zugrunde liegt [14, 18, 23], für o. g. Pathologien Raten von 0,7–3,6 % an, wohingegen diese bei retrospektiver Auswertung mit 6,1–10,4 % deutlich höher gefunden wurden [9, 20]. Sollten jedoch bis zu 40 % der Verschlüsse und hochgradigen Stenosen an den unmittelbar hirnzuführenden Arterien intraoperativ durch den Operateur zum Zeitpunkt der Sofortrekonstruktionsmöglichkeit nicht erkannt werden, lässt dies am Nutzen einer routinemäßig durchgeführten intraoperativen DSA zweifeln und könnte einer der Gründe sein, weshalb bisher kein Zusammenhang zwischen Sofortrekonstruktion und Outcome gezeigt werden konnte.

A.-carotis-externa-Pathologien wurden bei diesem Vergleich nicht berücksichtigt. Deren Bedeutung hinsichtlich einer möglichen Sofortkorrektur ist gefäßchirurgisch umstritten [2], weshalb ihr Auftreten keine prinzipielle Indikation zu einem Reeingriff darstellte. Das Nichtbeschreiben einer ACE-Pathologie durch den Operateur schien uns daher nicht zwangsläufig auf deren Nichtvorliegen schließen zu lassen, sondern könnte auch Folge deren Einschätzung als irrelevant gewesen sein. Diese Fehlermöglichkeit schien uns auch beim Vergleich der Ergebnisse vorangegangener Studien mit den Ergebnissen unserer Studie für Verschlüsse und hochgradige Stenosen der unmittelbar hirnzuführenden Arterien am geringsten zu sein.

Bei allen angiographisch detektierten Pathologien, intraoperativ oder im Rahmen der retrospektiven Auswertung, handelte es sich um Verschlüsse oder hochgradige Stenosen. Dies war insbesondere für die retrospektive DSA-Auswertung bemerkenswert, da diese formal und analog zur Auswertung der postoperativen Duplexsonographie erfolgte. Zwar lag die in unserer Untersuchung zur Anwendung kommende Kontrastmittel (KM)-Menge von 5–10 ml in der unteren Hälfte der in der Literatur beschriebenen [9, 15, 17, 18, 20, 22–24], die übrige technische Durchführung (30°-laterale als primäre, ggf. um weitere Projektionen ergänzte Aufnahme) war allerdings vergleichbar mit den meisten publizierten Studien. Auch macht der lange Beobachtungszeitraum mit Aktualisierung sowohl der angiographisch als auch duplexsonographisch zum Einsatz kommenden Geräte auf den jeweils zeitgemäßen Standard eine spezifische Gerätekonstellation als Ursache des Ergebnisses unwahrscheinlich.

Operativ induzierte hochgradige Stenosen und Verschlüsse sind für den betroffenen Patienten in Bezug auf eine Sofortrekonstruktion zwar die wichtigsten Befunde, geben aber dem Operateur vor dem Hintergrund der darüber hinaus zahlreichen duplexsonographisch detektierten operationstechnischen Unzulänglichkeiten keine für eine operationstechnische Qualitätskontrolle ausreichende Rückinformation. So wurden duplexsonographisch bei 10,3 % unserer Patienten im Operationsbereich an ACC oder ACI lokalisierte operationstechnische Unzulänglichkeiten beschrieben, was die angiographisch detektierte Rate von 2,3 % weit übertrifft und in dieser Form auch von anderen Autoren gesehen wurde [5, 18] und eine methodische Schwäche der Angiographie in dieser Situation nahelegt.

Interessanterweise waren dabei die primär am distalen Übergang des Patches zum nicht desobliterierten Gefäßsegment gefundenen Nahteinziehungen und Kinkings/Kalibersprünge mit einer linksseitigen TEA assoziiert (ausschließlich rechtshändige Operateure), was für ein in dieser Konstellation schwierigeres operatives Prozedere spricht. Der ebenfalls gefundene Zusammenhang mit einer Operation unter Verwendung eines (einschichtigen) Dacron-Patches gegenüber eines (dreischichtigen) PTFE-Patches könnte auf die geringere Steifigkeit des ersteren auf eine Fadenzugverformung zurückzuführen sein.

Den häufig beschriebenen Zusammenhang mit der Erfahrung des Operateurs konnten wir in unserem Kollektiv nicht nachweisen. Wir vermuten, dass dies zum einen statistische Gründe hat, so wurden knapp 80 % der Operationen von erfahrenen und sehr erfahrenen Operateuren durchgeführt, und zum anderen an unserem Status eines akademischen Lehrkrankenhauses liegt. So wurde ein unerfahrener, sich in der gefäßchirurgischen Ausbildung befindender Operateur von vorn herein nicht als Erstoperateur für eine Operation eingeplant, die bereits präoperativ als möglicherweise technisch kompliziert eingeschätzt wurde.

Zwar kommt nicht allen duplexsonographisch detektierten Pathologien eine gleich hohe Relevanz zu, Intima-Media-Ablösungen mitunter im Ausmaß eines Flaps, Nahteinziehungen und Kinkings/Kalibersprünge am Übergang des Patches zum nicht desobliterierten Gefäßabschnitt können aber nicht per se als klinisch irrelevant angesehen werden und stellen zu vermeidende operative Unzulänglichkeiten dar. Auch der fehlende Zusammenhang derartiger Pathologien mit dem klinisch-neurologischen Outcome in unserer Studie bzw. mit dem perioperativen Outcome allgemein [4, 9, 17, 23] erlaubt keine derartige Aussage, da hierbei nur Komplikationen bis 4 Wochen postoperativ eingehen. Dazu wäre eine longitudinale duplexsonographische und klinisch-neurologische Nachbeobachtung dieser Patienten erforderlich, die im Rahmen dieser Studie nicht erfolgten und bisher nur bei wenigen Untersuchungen zur intraoperativen Angiographie der Fall waren [10, 19], letztere mit zu wenigen Patienten, um auch eine mögliche Relevanz bei niedrigen Fallzahlen nachweisen zu können. Es überrascht daher nicht, dass wir in unserer Untersuchung „nur“ für Abstrompathologien und hier speziell für Thrombembolien – neben „erwarteten“ Prädiktoren wie Alter, Shunt und notfallmäßigem TEA-Eingriff – einen Zusammenhang mit einem perioperativen Schlaganfall und Tod gefunden haben.

Der Vergleich zwischen Angiographie und Duplexsonographie schloss neben ACE-Pathologien (Begründung s. oben) auch Pathologien im Abstrombereich aus. Letztere haben in der Mehrzahl der Fälle andere als operationstechnische Ursachen. In unserem Kollektiv handelte es sich bei allen Abstrompathologien um Thrombembolien und langstreckig enge Arteriensegmente. Erstere zeigten keinen Zusammenhang mit lokalen operationstechnischen Unzulänglichkeiten wie Nahteinziehungen, Intima-Media-Ablösungen und Kinkings/Kalibersprüngen, sehr wohl aber mit der allgemeinen (Ausmaß der Thrombozytenaggregationshemmung, perakute Schlaganfallsituation) wie auch lokalen Thrombogenität (höchstgradige Stenose mit vermindertem poststenotischem Flussvolumen). Langstreckig enge Arteriensegmente – ätiologieunabhängig als „langstreckig enges Gefäße“ beschrieben – dürften ihre Ursache zum einen in bereits präoperativ distal einer höchstgradigen Stenose engen (aber nicht pseudookklusiv veränderten) Gefäßen haben wie auch in einem intraoperativ auftretenden Vasospasmus. Beide Pathologien (Thrombembolien, langstreckig enge Gefäße) variierten im Zeitraum zwischen intraoperativer DSA und postoperativer Duplexsonographie beträchtlich, sodass der methodische Vergleich unter Einbeziehung dieser Pathologien zur Fehleinschätzung der Validität der einzelnen Untersuchungsmethoden geführt hätte. Aus diesem Grund wurden auch die intraoperativ sofort rekonstruierten TEA vom Methodenvergleich ausgeschlossen, da eine Befunddiskrepanz durch den zwischenzeitlich durchgeführten Korrektureingriff erklärt werden kann.

## Schlussfolgerung

Die Ad-hoc-Beurteilung der intraoperativen Kontrollangiographie im Anschluss an eine TEA geht mit einer nicht unerheblichen, in unserem Kollektiv 40 % ausmachenden Rate an übersehenen hochgradigen Stenosen und Verschlüssen in den unmittelbar hirnzuführenden Arterien (ACC, ACI, intrakranielle ACI, MCA) einher. Dies stellt ihren routinemäßigen Einsatz zur Erkennung sofort oder früh zu revidierender operativer Pathologien infrage und macht ihre alleinige Anwendung zu diesem Zweck obsolet. Die intraoperative DSA ist zur Detektion von i. d. R. nicht hochgradig stenosierenden operationstechnischen Unzulänglichkeiten methodisch nicht geeignet. Unabhängig von der Durchführung einer intraoperativen DSA sollte zur Qualitätskontrolle des operationstechnischen Ergebnisses postoperativ eine Duplexsonographie erfolgen, auch wenn die dabei detektierten operativen Unzulänglichkeiten wahrscheinlich mit einer sehr niedrigen Rate klinisch-neurologischer Komplikationen einhergehen.
